# Comparison of humoral immune response in heterologous and homologous COVID-19 booster vaccine groups using CoronaVac and mRNA-based BNT162b2 vaccines

**DOI:** 10.1590/0037-8682-0046-2023

**Published:** 2023-07-24

**Authors:** Serkan Atıcı, Ahmet Soysal, Erdem Gönüllü, Gökhan Aydemir, Naci Öner, Servet Alan, Havva Engin, Melek Yıldız, Metin Karaböcüoğlu

**Affiliations:** 1 Okan University School of Medicine, Division of Pediatric Infectious Diseases, İstanbul, Turkey. Okan University School of Medicine Division of Pediatric Infectious Diseases İstanbul Turkey; 2 Memorial Ataşehir Hospital, Division of Pediatric Infectious Diseases, İstanbul, Turkey. Memorial Ataşehir Hospital Division of Pediatric Infectious Diseases İstanbul Turkey; 3 Istanbul Health and Technology University, Department of Pediatrics, İstanbul, Turkey. Istanbul Health and Technology University Department of Pediatrics İstanbul Turkey; 4 Haliç University, Department of Pediatrics, İstanbul, Turkey. Haliç University Department of Pediatrics İstanbul Turkey; 5 Memorial Ataşehir Hospital, Clinic of Infectious Diseases, İstanbul, Turkey. Memorial Ataşehir Hospital Clinic of Infectious Diseases İstanbul Turkey; 6 Memorial Şişli Hospital, Clinic of Infectious Diseases, İstanbul, Turkey. Memorial Şişli Hospital Clinic of Infectious Diseases İstanbul Turkey; 7 Biruni University, Department of Pediatrics, İstanbul, Turkey. Biruni University Department of Pediatrics İstanbul Turkey

**Keywords:** COVID-19 vaccines, CoronaVac, BNT162b2, Booster dose, Heterologous vaccination schedule, Homologous vaccination schedule

## Abstract

**Background:**

Heterologous COVID-19 booster vaccination is an alternative strategy to homologous vaccination, especially in developing countries, due to shortages, delays, or unequal distribution of COVID-19 vaccines. We compared cohorts vaccinated with different vaccine combinations to investigate whether a heterologous booster dose of mRNA-based BNT162b2 vaccine boosts the immune response in individuals primed with the CoronaVac vaccine.

**Methods:**

Anti-RBD IgG is generally measured 4 weeks after primary immunization and 4 weeks after booster vaccination. Data on anti-receptor-binding domain (anti-RBD) IgG antibody titers and clinical characteristics were provided by infection control units.

**Results:**

The highest median anti-RBD IgG antibody titers (14589 AU/mL) after primary immunization was observed in the group vaccinated with two doses of BNT162b2 vaccine. Antibody titers were lower 4 months or more after the second CoronaVac vaccine dose in CoronaVac recipients with or without previous COVID-19. In the homologous COVID-19 booster vaccine group (primed with two doses of CoronaVac 4 weeks apart and a single booster dose of CoronaVac) the median anti-RBD titers decreased from 1025 to 242 AU/mL before the booster dose. In the heterologous group (primed with two doses of CoronaVac 4 weeks apart and a single booster dose of BNT162b2), the median anti-RBD titer increased to 31624 AU/mL, a 132-fold increase, 16 days after the booster dose.

**Conclusions:**

After the second dose of CoronaVac, protective neutralizing antibody levels decrease over time, and a booster dose is required. Heterologous COVID-19 booster vaccination with BNT162b2 is effective at boosting neutralizing antibody levels.

## INTRODUCTION

Between the identification of the first case of severe acute respiratory syndrome coronavirus 2 (SARS-CoV-2) infection in China in December 2019 and January 9, 2023, almost 650 million confirmed cases of coronavirus disease (COVID-19) and 6.6 million deaths were reported globally^1^. The Turkish Ministry of Health reported the first confirmed case of COVID-19 in Türkiye on March 11, 2020. In Türkiye, 17 042 722 confirmed cases of COVID-19 and 101 492 deaths were reported to the Turkish Health Authority by January 9, 2023, despite the Turkish government's strict pandemic control measures^2^. Vaccination is one of the most effective strategies for preventing the spread of SARS-CoV-2. Several vaccines have become available in different parts of the world; over 200 vaccine candidates have undergone preclinical studies and 40 have undergone human trials^3^. By July 25, 2022, more than 11.9 billion doses of COVID-19 vaccine had been administered worldwide^1^. The Turkish Health Authority COVID-19 vaccination program was initiated on January 11, 2021. Priority was given to healthcare workers (HCWs) and other high-risk groups. Two doses of CoronaVac 600 U/0.5 mL (Sinovac Life Science Co., Ltd., Beijing, China) were administered intramuscularly 4 weeks apart^4^. Once it became available on the Turkish market, the second COVID-19 vaccine, BNT162b2 (Pfizer-BioNTech), was added to the vaccination program with two doses administered 4 weeks apart^4^. Recently, the Turkish Ministry of Health approved a second inactive COVID-19 vaccine named TURCOVAC for emergency use in Türkiye^4^. Although mass vaccination programs have shaped COVID-19 pandemic curves, with the emergence of SARS-CoV-2 variants with accumulated mutations and waning immunity after two doses, concerns have been raised about the need for a booster dose of COVID-19 vaccine^5^. Therefore, many countries have implemented a booster dose of vaccine^6^. Since July 1, 2021, the Turkish Ministry of Health has offered a third (booster) dose of vaccine, with either the CoronaVac or BNT162b2 vaccine, at least 3 months after the second dose of COVID-19 vaccine^7^.

There is significant interest in heterologous prime-boost COVID-19 vaccination to prevent the depletion of stocks, which could reduce the worldwide supply of COVID-19 vaccines. In many countries it is recommended that people previously immunized with the ChAdOx1 nCoV-19 (Vaxzevria, AstraZeneca) vaccine should now receive a different a second dose with an alternative vaccine^8^. CoronaVac, an inactivated whole-virion vaccine has been widely used in a two-dose schedule. Multiple studies have assessed whether a third dose of the homologous vaccine or a different vaccine can boost the immune response^8^^-^^11^. Clemens et al.^9^ reported that antibody concentrations were low 6 months after immunization with two doses of CoronaVac. However, they observed that a third dose of the vaccine caused a significant increase in binding and neutralizing antibodies, which could increase protection against infection. They also reported that heterologous boosting resulted in more robust immune responses than homologous boosting and may enhance protection. Munro et al.^11^ investigated the reactogenicity and immunogenicity of seven different COVID-19 vaccines as a third dose to generate data for optimizing the selection of booster vaccines. They reported that all study vaccines boosted antibody and neutralizing responses after a ChAd/ChAd primary course, and except for a booster dose after a BNT162b2/BNT162b2 primary course, there were no safety concerns.

However, uncertainty remains regarding whether to select a homologous or a heterologous vaccine as the booster dose. There are currently insufficient data available on whether heterologous vaccine schedules can induce robust cellular and humoral responses. Mixed vaccination schedules may induce stronger humoral and cellular immune responses against a range of SARS-CoV-2 variants. The primary objective of this study was to compare the humoral immune responses of individuals who received heterologous (booster dose with mRNA-based BNT162b2 in addition to two doses of CoronaVac) and homologous (only CoronaVac or BNT162b2 vaccine doses) vaccination schedule.

## METHODS

**FIGURE 1:**
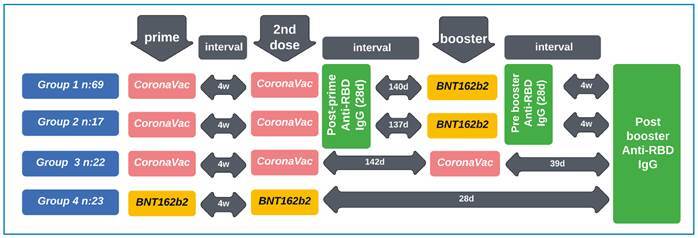
Study timeline and group characteristics.

This study was conducted at Memorial Ataşehir and Şişli Hospitals in İstanbul, Türkiye, between July 1 and August 31, 2021. In this retrospective observational cohort study, we compared different vaccination combinations to analyze humoral immunological responses elicited by heterologous and homologous vaccination schedules. Participants were questioned about their history of confirmed or suspected SARS-CoV-2 infection, and their consent was obtained. In addition, the number of doses of CoronoVac or BNT162b2 vaccine was determined, and participants were divided into four separate vaccination cohorts according to the vaccines administered to the participants. Group 1 consisted of individuals who did not report any previous confirmed or suspected SARS-CoV-2 infection who were primed with two doses of CoronaVac 4 weeks apart, and received a single booster dose of BNT162b2 vaccine. Group 2 consisted of individuals with confirmed previous SARS-CoV-2 infection who were primed with two doses of CoronaVac 4 weeks apart and received a single booster dose of BNT162b2. Group 3 consisted of individuals who did not report any previous confirmed or suspected SARS-CoV-2 infection who were primed with two doses of CoronaVac 4 weeks apart and received a single booster dose of CoronaVac. Group 4 consisted of individuals who did not report any previous confirmed or suspected SARS-CoV-2 infection who received two doses of BNT162b2, 3 to 4 weeks apart. The study timeline is shown in Figure 1**.**

Data on the anti-receptor-binding domain (anti-RBD) immunoglobulin G (IgG) antibody titers and clinical characteristics of participants were provided by the infection control units of the two study hospitals. Anti-RBD IgG is generally measured 4 weeks after primary immunization and 4 weeks after booster vaccination. The SARS-CoV-2 IgG II Quant Reagent Kit (Abbott Ireland Diagnostics Ltd., Sligo, Ireland) was used to detect antibody levels against the RBD of the SARS-CoV-2 spike protein. This study was approved by the Memorial Şişli Hospital ethics committee and The Turkish Ministry of Health scientific research commission. Statistical analyses were performed using Jamovi (version 1.6; computer software retrieved from https://jamovi. org). Antibody titers between groups were tested using the Student’s t-test or 2-tailed Mann-Whitney-U tests for continuous variables, and , Pearson χ^2^ test for categorical variables.

## RESULTS

Of the 131 participants enrolled in the study, 108 (82%) were HCWs. In the study cohort, 17 HCWs (16%) had laboratory-confirmed mild or asymptomatic previous SARS-CoV-2 infection, whereas 91 (84%) HCWs had no previous history of infection. SARS-CoV-2 infection was diagnosed based on a positive polymerase chain reaction (PCR) test results of a nasopharyngeal swab. The remaining 23 (18%) individuals were healthy previously uninfected adults. The demographic and immunization characteristics of the study population are shown in Table 1.

**TABLE 1:** Demographic and immunization characteristics of the study groups.

	Group 1	Group 2	Group 3	Group 4	Total
**Number of individuals (%)**	69 (52.7)	17 (12.9)	22 (16.8)	23 (17.6)	131 (100)
**Males (%)**	22 (50)	4 (9.1)	10 (22.7)	8 (18.2)	44 (100)
**Females (%)**	47 (54.1)	13 (14.9)	12 (13.8)	15 (17.2)	87 (100)
**Median age (range)**	35 (22-71)	36 (22-62)	38 (22-52)	36 (30-45)	36 (22-71)
**Primary immunization**	2 doses CoronaVac	2 doses CoronaVac	2 doses CoronaVac	2 doses BNT162b2	2 doses of vaccine
**Booster dose**	BNT162b2	BNT162b2	CoronaVac	None	0 or 1 dose of vaccine
**Previous SARS-CoV2 infection history**	None	Yes	None	None	

### Comparison of anti-RBD IgG antibody titers after primary immunization

The highest median anti-RBD IgG antibody titers after primary immunization were observed in Group 4 (14589 AU/mL), the second highest median levels were in Group 2 (median:1697 AU/mL), and the third highest median levels were in Group 1 (median: 1025 AU/mL) (Table 2). The median anti-RBD IgG antibody titer in Group 4 was significantly higher than that in Groups 1 and 2 (p < 0.001). However, the median anti-RBD IgG antibody titers did not differ between Groups 1 and 2 (p = 0.99). None of the Group 3 members had their anti-RBD IgG antibody titers measured after primary immunization.

**TABLE 2:** Immunization and anti-RBD IgG antibody titer characteristics of the study groups.

	Group 1	Group 2	Group 3	Group 4
**Primary immunization**	2 doses CoronaVac	2 doses CoronaVac	2 doses CoronaVac	2 doses BNT162b2
**Booster immunization**	Single dose BNT162b2	Single dose BNT162b2	Single dose CoronaVac	None
**Previous SARS-CoV-2 infection history**	None	**YES**	None	None
**Mean antibody titers after primary immunization (AU/mL)**	1384 [1054] (85-4605)	1806 [946] (528-3451)	Not done	17781 [10638] (3777-377700)
**Median antibody titers after primary immunization (AU/mL)**	1025	1697	Not done	14589
**Median days of antibody level measurement after primary immunization**	28 (16-56)	29 (24-35)	Not done	28 (14-55)
**Median Pre-booster dose antibody titers (AU/mL)**	242 (50-7785)	1450 (154-23330)	Not done	No booster dose
**Median Post-booster antibody titers (AU/mL)**	35834 (2030-40000)	39492 (6201-40000)	2354 (104-24448)	No booster dose
**Median time Post-booster antibody titers measurements (days)**	16 (12-53)	16 (12-19)	19 (14-35)	No booster dose
**The median interval between 2 and booster doses of vaccine (days)**	140 (119-157)	137 (114-168)	142 (140-143)	No booster dose

Kinetics of anti-RBD IgG antibody titers after two doses of CoronaVac primary immunization boosted with a single dose of BNT162b2 vaccine

We evaluated the anti-RBD IgG levels in Group 1 at least 131 days after participants had received the two primary doses of CoronaVac. The median anti-RBD titer decreased from 1025 to 242 AU/mL before the booster dose. A single booster dose of BNT162b2 was administered a median of 140 days later. Sixteen days after the booster dose, the median anti-RBD titer increased to 35834 AU/mL, a 148-fold increase (p < 0.001). Anti-RBD IgG levels were evaluated members of Group 2 (with previous SARS-CoV-2 infection) at least 114 days after completing the two doses of CoronaVac. The median anti-RBD titer decreased from 1697 to 1450 AU/mL before the booster dose. A single booster BNT162b2 was administered 137 days later. Sixteen days after the booster dose, the median anti-RBD titer increased to 39492 AU/mL, a 27-fold increase (p < 0.001). The immunization and anti-RBD IgG antibody titer characteristics of the study groups are shown in Table 2.

### Comparison of anti-RBD IgG antibody titers after vaccination with CoronaVac or BNT162b2

**FIGURE 2:**
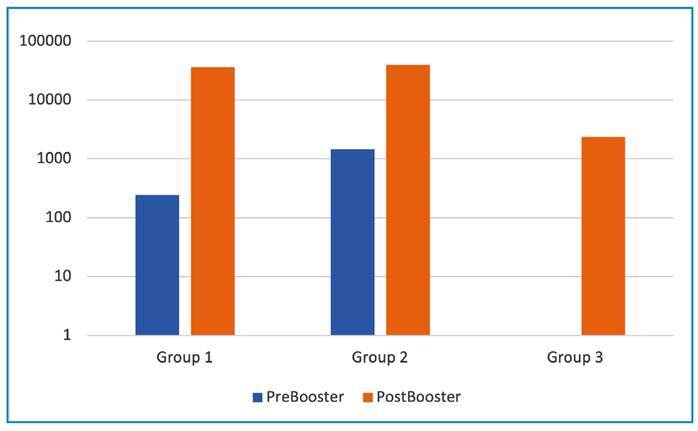
Pre-and post-booster anti-RBD antibody titers (AU/mL) between study groups as logarithmic scale.

In Group 1, the median anti-RBD IgG titer, was 242 AU/mL before the booster dose, and increased 130-fold to 35834 AU/mL after a single booster dose of BNT162b2 (p < 0.001). In Group 2 (previous SARS-CoV-2 infection), the median anti-RBD IgG titer, which was 1450 AU/mL before the booster dose, and increased 27-fold to 39492 AU/mL after a single booster dose of BNT162b2 (p = 0.008). The pre- and post-booster anti-RBD antibody titers of each study group are shown in Figure 2. None of the Group 3 members had their anti-RBD IgG antibody titer measured before receiving a booster dose, but their anti-RBD IgG antibody titers were measured at least 14 days after a single booster dose of CoronaVac Of the 22 participants in Group 3 with anti-RBD IgG antibody titer results, the median titer was 2354 AU/mL after the booster dose (Figure 2). There were no statistically significant difference between Groups 1 and 2 (p = 0.89). However, Groups 1 and 2 had significantly higher median anti-RBD IgG antibody titers than Group 3 (p<0.001). Group 1 had a 15-fold higher median anti-RBD IgG antibody titer than Group 3; and Group 2 had 17-fold higher median anti-RBD IgG antibody titer than Group 3. We compared total anti-RBD antibody levels for different vaccination schedules. The highest anti-RBD values were observed in Group 2, followed by Group 1, and the lowest in Group 3. Two doses of BNT162b2 (Group 4) elicited a higher antibody titer than a homologous CoronaVac-primary vaccination and CoronaVac- booster dose (Group 3).

## DISCUSSION

This study compared the humoral immunogenicity of BNT162b2 and CoronaVac as booster vaccination doses in in individuals with and without previous SARS-CoV-2 infection. In addition, anti-RBD IgG antibody titers were monitored longitudinally after two doses of each vaccine and before and after the booster dose. 

The first finding of this study is that primary immunization with the BNT162b2 vaccine elicited a higher anti-RBD IgG antibody response than that elicited by the CoronaVac vaccine. The median anti-RBD IgG antibody titers in participants who received two doses of the BNT162b2 vaccine group was 14-fold higher than those who received two doses of CoronaVac without previous SARS-CoV-2 infection, and ten-fold higher than those who received two doses of CoronaVac with previous SARS-CoV-2 infection. BNT162b2 primary and booster vaccines are superior to CoronaVac primary and booster vaccines in terms of increasing the anti-RBD titer. This difference was not solely due to the heterologous booster approach because the homologous primary BNT162b2 series produced a more robust response than the homologous Coronavac booster. The median anti-RBD IgG antibody titers of the group that received the BNT162b2 booster (Group 4) were significantly higher than those of Groups 1 and 2. According to recent studies, the immune response after CoronaVac is significantly lower than that after BNT162b2, although it is above the threshold value thought to be protective^9^^,^^11^^-^^14^. Mok et al.^12^ reported that BNT162b2 elicited significantly higher 50% and 90% plaque reduction neutralization (PRNT50 and PRNT90), spike FcR-binding, spike S2 domain-binding, spike N-terminal domain-binding, spike receptor-binding, surrogate virus neutralization test (sVNT) and antibody avidity than CoronaVac one month after the second dose. Studies conducted with both vaccines have consistently shown that the immune response after two doses of CoronaVac is significantly lower than that after two doses of BNT162b2. Sauré et al.^13^ reported a significantly higher rapid test kit SARS-CoV-2 IgG positivity in BNT162b2 recipients than in CoronaVac recipients 1 to 4 weeks after the first dose and 5 to 9 weeks after the second dose of vaccine in a study conducted in Chile. Lim et al.^14^ reported significantly higher PRNT50 and PRNT90 geometric mean titers after two doses of BNT162b2 than after two doses of CoronaVac in a cohort of 93 HCWs in Hong Kong.

The second finding is that antibody responses decreased from 4 months after the second dose of CoronaVac both in CoronaVac recipients with and without previous SARS-CoV-2 infection. Four months or more after the second dose of CoronaVac, the median anti-RBD IgG antibody titers decreased four-fold in previously infected HCWs, and three-fold HCWs without previous infection. None of the anti-RBD IgG antibody titers decreased to the lower limit of the detection threshold, regardless of whether They had a history of SARS-CoV-2 infection. Fonseca et al.^15^ reported a significantly decreased median anti-S IgG level 6 months after the second dose of CoronaVac in HCWs, and Balkan et al.^16^ reported that humoral immune response after two doses of CoronaVac administration decreased with time and that a booster dose was required.

In this study, we detected an adequate anti-RBD IgG response with administration of either a homologous or heterologous booster dose of vaccine after the second dose of the CoronaVac vaccine. Previous studies have generally found that the antibody response is lower and the prevalence of seronegativity is higher with CoronaVac than with other vaccine types^13^^-^^16^. However, the clinical relevance of this reduction should be carefully considered, as circulating antibody titers are not necessarily predictive of T-cell memory^17^.

Chen et al.^18^ assessed the antibody dynamics of a homologous booster after two-primary doses of CoronaVac in younger and older adults. Neutralizing antibodies decreased 6 to 8 months after the second dose of vaccine, four-fold in younger adults, and 15-fold in older adults. Moreover, a 17-fold increase and 40-fold increase in the neutralizing antibody titer was observed in younger and older adults, respectively, 15 days after a booster dose. Studies that used heterologous mRNA or adenoviral vector-based vaccines as booster doses after the first two doses of CoronaVac have found that the post-booster antibody response was significantly higher than after a homologous CoronaVac booster dose^13^^,^^19^^-^^21^. In a sentinel study of CoronaVac and BNT162b2 vaccines, 4 weeks after one dose, the prevalence of IgG seropositivity was lower among CoronaVac recipients than among BNT162b2 recipients 4 weeks after the first dose (28.1% vs. 79.4%) and 3 weeks after the second dose (77.4% vs. 96.5%). Furthermore, a stable decrease in IgG titers was observed in the CoronaVac recipients 4 to 16 weeks after the second dose, in contrast to the findings in the BNT162b2 vaccine group^13^.

Although the CoronaVac and AstraZeneca vaccines are highly protective, a cross-sectional survey revealed a similar tendency for the serum antibody levels to decrease after the second dose. In addition, serum levels of specific antibodies to SARS-CoV-2 spike protein and nucleoprotein were low 4 to 6 months after the second vaccine dose^19^.

There is growing interest in the efficacy of heterologous COVID-19 vaccination strategies owing to potential vaccine availability problems, questions about protection against emerging variant viruses, and variable vaccine schedules in different countries. In addition, heterologous vaccination strategies, such as inactivated vaccines followed by a viral vector-based vaccine or a vector-based vaccine followed by an mRNA vaccine, may provide a stronger immune response and good tolerability compared with a homologous vaccine schedule^20^^-^^21^.

In December 2021, the World Health Organization issued an interim statement about considering heterologous COVID-19 vaccination schedules. The statement advised that mRNA- or vector-based vaccines can be considered instead of an inactivated vaccine for a third vaccine dose in those who have received two initial doses of inactivated vaccine^22^. After two inactivated vaccine doses, the mRNA booster schedule could augment neutralization activity, specific antibody levels, and memory T and B cell responses against Omicron variants of and other SARS-CoV-2 variants of concern. Therefore, a heterologous prime-boosting schedule may be a promising vaccination strategy^23^^-^^25^. We observed both a steady decrease in antibody titers after the second dose of CoronaVac and increased antibody titers with the heterologous BNT162b2 booster after the second dose of CoronaVac in uninfected HCWs (Group 1) and infected HCWs (Group 2). Other studies have shown that a heterologous dose of mRNA vaccine after CoronaVac can promote a strong antibody response. In a study conducted in Brazil, Clemens et al.^26^ found that the immune response after two doses of CoronaVac was boosted after by a third heterologous dose of a recombinant adenoviral vector vaccine (AZD1222, AstraZeneca or Ad26.COV2-S, Janssen), or an mRNA vaccine (BNT162b2, Pfizer-BioNTech) vaccine. In contrast, a third homologous dose of CoronaVac produced weaker immune response. Filardi et al.^27^ reported that IgG titers against SARS-CoV-2 RBD or spike protein and neutralization titers against Omicron sublineages were decreased in individuals who received homologous CoronaVac compared with those who received a heterologous BNT162b2 or ChAdOx1 booster.

Although antibody levels 6 months after completing two doses of CoronaVac appears to decrease near or below the lower limit of seropositivity, a third homologous dose of CoronaVac administered 8 months after the second dose is associated with a three- to five-fold increase in detectable neutralizing antibodies^10^.

This study has some limitations. First, it is a small longitudinal cohort study representing a limited number of HCWs in Türkiye during the study period. Despite the relatively small sample size, we found statistically significant differences between the vaccination groups. Second, it is still uncertain how these results compare to the overall antibody levels induced by the BTN162b2 and CoronaVac vaccines in the general population. Hence, more extensive studies are required to draw definitive conclusions. In addition, T-cell responses were not assessed in our study. Therefore, serum inflammatory markers and pro-inflammatory cytokine levels, such as tumor necrosis factor alpha, interleukin 6 (IL-6), IL-2, and cytotoxic T cells, should be compared with the humoral immune response in future studies.

Third, we did not have data to determine the level of pre-booster antibodies in the homologous CoronaVac booster group (Group 4) to compare the dynamics of homologous pre-and post-booster. In this study, a decrease was observed in the anti-RBD IgG titer in the long term after the second dose of CoronaVac in the other CoronaVac-primed study groups (Groups 1 and 2). However, recent studies focusing on the CoronaVac immune response dynamics have shown a steady decrease in anti-RBD IgG and a rapid increase after a homologous dose^13^^,^^26^. Fourth, we did not assess the safety of booster vaccines; however, previous studies have demonstrated the safety of booster doses of CoronaVac. Clemens et al.^9^ investigated immune responses of either homologous CoronaVac or heterologous adenoviral-vector vaccine (Ad26.COV2-S, Janssen), an mRNA vaccine (BNT162b2, Pfizer-BioNTech), and a recombinant adenoviral-vector vaccine (AZD1222, AstraZeneca) in healthy adults in Brazil after completing two doses of CoronaVac. Deficient neutralizing antibody titers were detected 6 months after two doses of CoronaVac. Both homologous and heterologous COVID-19 booster vaccinations are safe and strongly enhance humoral immune responses. Li et al.^28^ investigated the safety and immunogenicity of homologous CoronaVac and heterologous AD5-nCOV plus CoronaVac booster immunization after two doses of CoronaVac. Only mild adverse reactions (4.0-4.8%) were observed in the CoronaVac group. No severe adverse reactions were observed in the CoronaVac group^28^. Fifth, the study had a predominance of HCWs (82%), so the results might not be generalizable to the general population, given that the immune response in HCWs may differ from that of the general population^29^.

In conclusion, our study is one of a limited number of studies showing the dynamics of the vaccine response after two doses of CoronaVac both in individuals with and without previous SARS-CoV-2 infection , and to describe the immune response to homologous and heterologous booster doses. Although immune response studies in healthy individuals are abundant during phase 3 and 4 studies of vaccines, there are relatively few published studies related to CoronaVac. This study could contribute to the development of heterologous vaccine programs and practices. In conclusion, a growing body of evidence shows that after the second dose of CoronaVac, protective neutralizing antibody levels decrease over time and a booster dose is required. This study showed that heterologous COVID-19 booster vaccination with BNT162b2 after two doses of CoronaVac is effective at boosting neutralizing antibody levels.
